# The safety of perioperative dexamethasone with antiemetic dosage in surgical patients with diabetes mellitus: a systematic review and meta-analysis

**DOI:** 10.1186/s13741-023-00293-4

**Published:** 2023-03-08

**Authors:** Qian-Yun Pang, Jing-Yun Wang, Xiao-Long Liang, Yan Jiang, Hong-Liang Liu

**Affiliations:** 1 Department of Anesthesiology, Chongqing University Cancer Hospital, No. 181, Hanyu Road, Shapingba District, Chongqing City, 400030 China; 2 School of Medicine, Chongqing University; Chongqing University Cancer Hospital, Chongqing, 400030 China

**Keywords:** Dexmethasone, Antiemesis, Diabetes mellitus, Surgery, Glucose, Wound healing

## Abstract

**Background:**

Dexamethasone is commonly used for antiemesis in surgical patients. It has been confirmed that long-term steroid use increases blood glucose level in both diabetic and non-diabetic patients, it is unclear how a single dose of intravenous dexamethasone used pre/intraoperatively for postoperative nausea and vomiting (PONV) prophylaxis would influence the blood glucose and wound healing in diabetic patients.

**Methods:**

The Pubmed, Cochrane Library, Embase, Web of Science databases, CNKI and Google Scholar were searched. The articles reporting a single dose dexamethasone administered intravenously for antiemesis in surgical patients with diabetes mellitus (DM) were included.

**Results:**

Nine randomized controlled trials (RCTs) and 7 cohort studies were included in our meta-analysis. The results showed that dexamethasone increased glucose level intraoperatively (MD: 0.439, 95% CI: 0.137–0.581, *I*^2^ = 55.7%, *P* = 0.004), at the end of surgery (MD: 0.815, 95% CI: 0.563–1.067, *I*^2^ = 73.5%, *P* = 0.000), on postoperative day (POD) 1 (MD: 1.087, 95% CI: 0.534–1.640, *I*^2^ = 88%, *P* = 0.000), on POD 2 (MD: 0.501, 95% CI: 0.301–0.701, *I*^2^ = 0%, *P* = 0.000), and increased peak glucose level within 24 hours of surgery (MD: 2.014, 95% CI: 0.503–3.525, *I*^*2*^ = 91.6%, *P* = 0.009) compared with control. It indicated that dexamethasone caused the increase of perioperative glucose level at different time points by 0.439 to 1.087 mmol/L (7.902 to 19.566 mg/dL), and the increase of peak glucose level within 24 hours of surgery by 2.014 mmol/L (36.252 mg/dL) compared with control. Dexmethasone had no impact on wound infection (OR: 0.797, 95%CI: 0.578–1.099, *I*^2^ = 0%, *P* = 0.166) and healing (*P* < 0.05).

**Conclusion:**

Dexamethasone could increase blood glucose by only 2.014 mmol/L (36.252 mg/dL) of peak glucose level within 24 hours of surgery in surgery patients with DM, the increase of glucose level at each time point perioperatively was even lower, and had no effect on wound healing. Thus, dexamethasone with a single dose could be safely used for PONV prophylaxis in diabetic patients.

**Trial registration:**

The protocol of this systematic review was registered in INPLASY with the registration number INPLASY202270002.

**Supplementary Information:**

The online version contains supplementary material available at 10.1186/s13741-023-00293-4.

## Introduction

Dexamethasone is commonly used and very effective to prevent postoperative nausea and vomiting (PONV), and dexamethasone combined with 5-HT3 receptor antagonist is recommended as the first-line antiemetic for children (Gan et al. 2020). Long-term steroid use increases blood glucose level in both diabetic and non-diabetic patients (Perez et al. 2014a; Shah et al. 2022; Suh and Park 2017). A single dose of dexamethasone in non-cardiac surgery can increase blood glucose till 24 hours after surgery (Perez et al. 2014b). One recent meta-analysis showed that dexamethasone significantly increased blood glucose in non-diabetic surgical patients compared with control within 12 hours of surgery (Polderman et al. 2018). With the global rising of diabetes mellitus (DM), it is unclear how a single dose of intravenous dexamethasone used pre/intraoperatively for PONV prophylaxis would influence the blood glucose and wound healing in diabetic patients.

Therefore, we conducted a meta-analysis in this study to compare the blood glucose changes, and wound healing in surgical patients with DM between a single dose of dexamethasone and no usage, and tried to confirm the safety of dexamethasone use for PONV prophylaxis in diabetic patients.

## Materials and methods

### Protocol and registration

We conducted this meta-analysis in accordance with 2020 PRISMA (Preferred Reporting Items for Systematic Reviews and Meta-Analyses) and MOOSE (Meta-Analyses and Systematic Reviews of Observational Studies in Epidemiology) guidelines. The protocol of this systematic review was registered in INPLASY with the registration number INPLASY202270002.

### Inclusion and exclusion criteria

The inclusion criteria included: (a) studies comparing dexamethasone perioperatively with control (no dexamethasone treatment or saline) in adult patients with diabetes mellitus undergoing non-cardiac surgery; (b) dexamethasone with a single dose administered intravenously for antiemesis; (c) data about the relationship between dexamethasone and glucose responses with or without wound healing and infection. The exclusion criteria included: (a) studies comparing different doses of dexamethasone without control; (b) dexamethasone administered only postoperatively; (c) dexamethasone with two or more doses for antiemesis; (d) dexamethasone with large dosage for other purposes (not for antiemesis); (e) conference, review or case report.

### Search strategy and outcome

Two researchers independently (Y. J. and J.W.) searched the databases of Pubmed, Embase, Cochrane Library, Web of science, CNKI, and Google Scholar from 1990 to June 2022, and screened the articles. The key words and medical subject headings (MeSH) included dexamethasone, steroids, surgery, surgical, operation, operative, diabetes mellitus, diabetic, diabetes, antiemesis, antiemetic, emesis, emetic, postoperative nausea and vomiting, PONV, glucose, glucose response, adverse events, wound infection and wound healing. The detailed search strategy was shown in Supplementary file 1. References in the retrieved literature were also checked to determine any potential eligible trials. The primary outcome was glucose responses, which included glucose changes after dexamethasone administration, and peak glucose level. The secondary outcomes included insulin requirement, wound healing and infection.

### Data extraction and quality assessment

Two researchers (Q.P. and X.L.) independently assessed the quality of each included articles and extracted the data according to the predefined inclusion and exclusion criteria. Any discrepancy was resolved by a consensus process involving a third author (H.L.). The following data were extracted from each article: the author, publication year, study design, allocated groups, sample size, intervention, and outcomes. When the data were presented as median or graphs, we tried to contact the corresponding author by email for the raw data, if no response, we then transferred the data from median to mean ± SD (Hozo et al. 2005), or transformed the data from graph to numbers using Plot-digitizer software (version 4.1, Mitchell, http://markummitchell.github.io/engauge-digitizer/).

The quality of RCTs was assessed using the modified Jadad scoring system. The evaluated items included size calculation, generation of allocation sequence, allocation concealment, methods of randomization, blinding, and descriptions of protocol deviations, withdrawals, and dropouts. The highest score was 7, and the trial with a quality score less than 3 was excluded. The quality of the observational studies was assessed using the Newcastle-Ottawa Scale (NOS). The evaluated items included selection criteria, comparability and outcome (cohort) or exposure (case-control). The maximum score was 9, and the trial with a score less than 7 was excluded.

Grading of Recommendations, Assessment, Development and Evaluation (GRADE) methodology was used to appraise the overall evidence- based quality of each outcome.

### Statistics analysis

The meta-analysis was conducted using Stata software (version 14). The calculation of effect size for blood glucose was mean differences (MD) with 95% confidential index (CI), the units of glucose were mmol/L and mg/dL, and in the forest plot, the units of glucose level was presented as mmol/L. The MD value of glucose levels at different time points between dexamethasone and control, and the MD value of peak glucose level within 24 hours of surgery between dexamethasone and control represent the magnitude of blood glucose rise caused by dexamethasone. The calculation of effect size for insulin requirement was standardized mean differences (SMD) with 95% CI. The effect size for dichotomous data was presented as odds ratio(OR) with 95% CI. The data of glucose levels at each time point (pre/intra/postoperatively) were expressed as mean, and the trend of glucose changes was presented. The between-study heterogeneity was determined by *I*^2^ value, the levels of heterogeneity were defined as low when *I*^2^ ≤ 25%, moderate when *I*^2^ ranges 25–50%, and high when *I*^2^ > 50%. The fixed-effect model was used when *I*^2^ ≤ 50%, and the random-effect model was used when *I*^2^ > 50%. Subgroup analysis and sensitivity analysis were performed to explore the source and size of heterogeneity among studies when necessary. The egger test was used for publication bias evaluation, and there was no statistical publication bias if *P* > 0.05 from egger test.

## Results

### Literature search and retrieval

**Fig. 1:**
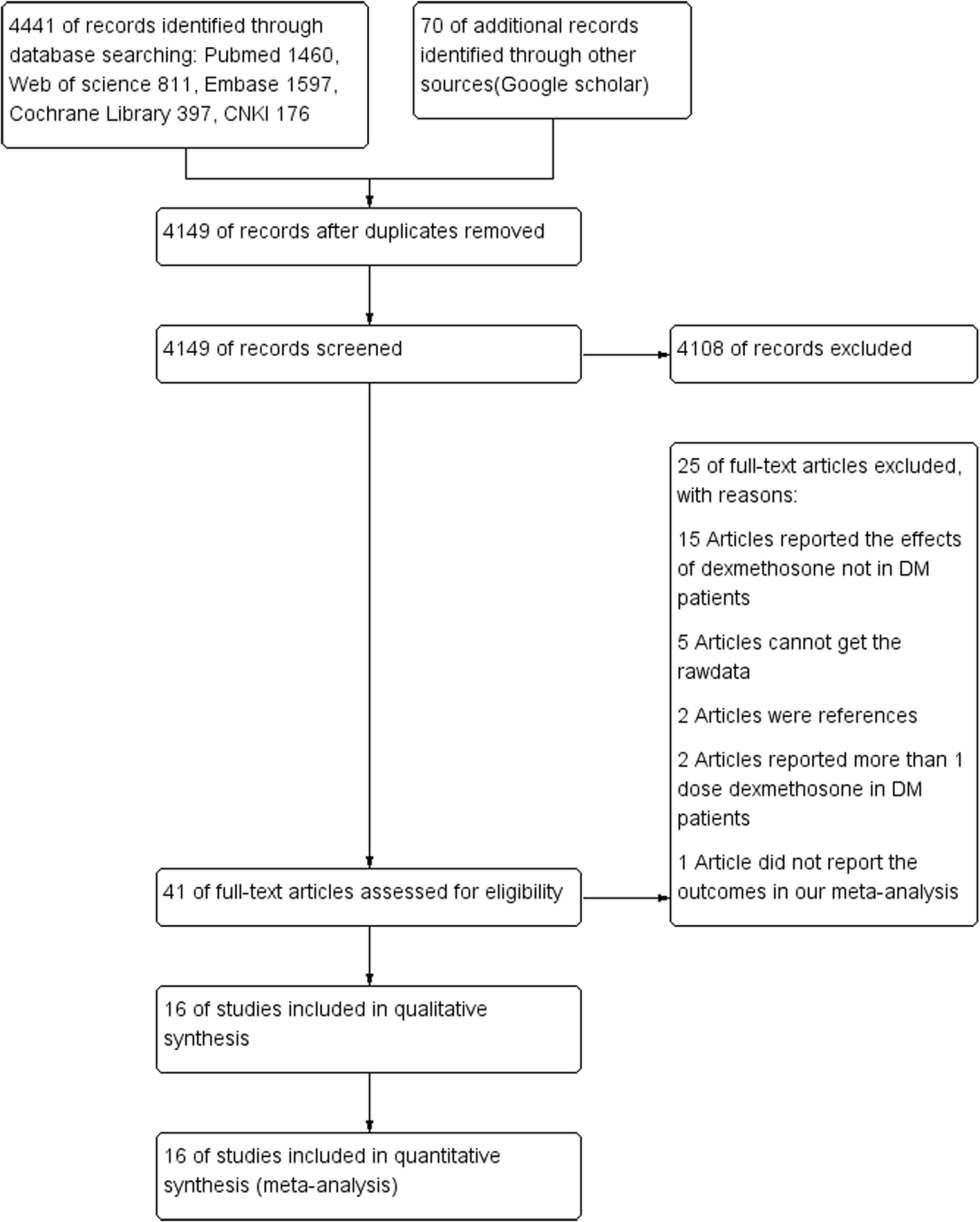
Flowchart of studies screening

Our search strategy identified 4511 citations from the databases and other sources. The full versions of 41 articles were retrieved after screening and conducting a detailed selection process. Finally 16 articles (Nazar et al. 2011; Tien et al. 2016; Nazar et al. 2009; Corcoran et al. 2021a; Backes et al. 2013; Purushothaman et al. 2018; Zhang et al. 2014; Shang et al. 2022; Wasfie et al. 2018; Herbst et al. 2020; Godshaw et al. 2019; Allen et al. 2020; Egan et al. 2019; Harding et al. 2021; O'Connell et al. 2018; Corcoran et al. 2021b) met the inclusion criteria and underwent data extraction. The details of the screening procedure are shown in Fig. 1.

### Study characteristics

The 16 included studies (Nazar et al. 2011; Tien et al. 2016; Nazar et al. 2009; Corcoran et al. 2021a; Backes et al. 2013; Purushothaman et al. 2018; Zhang et al. 2014; Shang et al. 2022; Wasfie et al. 2018; Herbst et al. 2020; Godshaw et al. 2019; Allen et al. 2020; Egan et al. 2019; Harding et al. 2021; O'Connell et al. 2018; Corcoran et al. 2021b) were published from 2009 to 2022, 9 were RCTs (Nazar et al. 2011; Tien et al. 2016; Nazar et al. 2009; Corcoran et al. 2021a; Backes et al. 2013; Purushothaman et al. 2018; Zhang et al. 2014; Shang et al. 2022; Corcoran et al. 2021b), and the other 7 (Wasfie et al. 2018; Herbst et al. 2020; Godshaw et al. 2019; Allen et al. 2020; Egan et al. 2019; Harding et al. 2021; O'Connell et al. 2018) were cohort studies, all studies reported dexamethasone for antiemesis, and the dosage of dexamethasone ranged from 4 to 12 mg daily during the perioperative period. In the 9 RCTs, dexamethasone was administered at induction of anesthesia or before anesthesia, 8 studies (Nazar et al. 2011; Tien et al. 2016; Nazar et al. 2009; Corcoran et al. 2021a; Purushothaman et al. 2018; Zhang et al. 2014; Shang et al. 2022; Corcoran et al. 2021b) compared dexamethasone with saline, and 1 (Backes et al. 2013) compared dexamethasone with no treatment. In the 7 cohort studies, dexamethasone was administered intraoperatively in 4 studies (Wasfie et al. 2018; Egan et al. 2019; Harding et al. 2021; O'Connell et al. 2018), perioperatively in 1 study (Herbst et al. 2020), and in 2 studies (Godshaw et al. 2019; Allen et al. 2020) at induction of anesthesia. All these 7 cohort studies compared dexamethasone with no treatment. The detailed characteristics and quality assessment for each article were presented in Table 1.

**Table 1:** The characteristics for included articles

Author	Publication time	Design	Groups	Intervention	Outcomes	Jadad/ NOS score
Tien et al. (Tien et al. 2016)	2016	RCT	Dex (*n* = 20)	Dex 8 mg at induction	Maximal glucose change, glucose levels, insulin required	7
			C (*n* = 24)	Ondansetron 4 mg at induction		
Nazar et al. (Nazar et al. 2009)	2009	RCT	Dex (*n* = 15)	Dex 8 mg at induction	Maximal glucose change, glucose levels	4
			C (n = 15)	Saline at induction		
Corcoran et al. (Corcoran et al. 2021a)	2020	RCT	Dex (4) (*n* = 22)	Dex 4 mg at induction	Maximal glucose change, glucose levels, wound infection and dehiscence	5
			Dex (8) (*n* = 19)	Dex 8 mg at induction		
			C (n = 22)	Saline at induction		
Backes et al. (Backes et al. 2013)	2013	RCT	Dex (*n* = 41)	Dex 10 mg at induction	Maximal glucose change, maximal glucose level	6
			C (*n* = 37)	No treatment		
Nazar et al. (Nazar et al. 2011)	2011	RCT	Dex (n = 15)	Dex 8 mg at induction	Maximal glucose, glucose levels	4
			C (n = 15)	Saline at induction		
Purushothaman et al. (Purushothaman et al. 2018)	2018	RCT	Dex (4) (*n* = 45)	Dex 4 mg pre-anesthesia	Maximal glucose, glucose levels	4
			Dex (8) (n = 45)	Dex 8 mg pre-anesthesia		
			C (n = 45)	Saline pre-anesthesia		
Zhang et al. (Zhang et al. 2014)	2014	RCT	Dex (*n* = 10)	Dex 10 mg pre-anesthesia	Maximal glucose change, glucose levels	3
			C (n = 10)	Saline pre-anesthesia		
Shang et al. (Shang et al. 2022)	2022	RCT	Dex (n = 45)	Dex 0.11 mg/kg post-induction	Glucose levels, insulin required, wound infection and dehiscence	4
			C (n = 45)	Saline post-induction		
Corcoran et al. (Corcoran et al. 2021b)	2021	RCT	Dex (*n* = 583)	Dex 8 mg at induction	Wound infection	7
			C (*n* = 571)	Saline at induction		
Wasfie et al. (Wasfie et al. 2018)	2018	Cohort study	Dex (*n* = 119)	Dex intraoperative	Glucose levels	7
			C (*n* = 235)	No treatment		
Herbst et al. (Herbst et al. 2020)	2020	Cohort study	Dex (*n* = 626)	Dex perioperative	Maximal glucose, glucose levels	7
			C (*n* = 520)	No treatment		
Godshaw et al. (Godshaw et al. 2019)	2019	Cohort study	Dex (*n* = 428)	Dex 6 or 12 mg pre-incision	Glucose levels, infection	6
			C (*n* = 229)	No treatment		
Allen et al. (Allen et al. 2020)	2020	Cohort study	Dex (*n* = 92)	Dex 8 mg pre-incision	Maximal glucose,glucose levels, insulin required	7
			C (*n* = 193)	No treatment		
Egan et al. (Egan et al. 2019)	2019	Cohort study	Dex (*n* = 17)	Dex 4 or 10 mg intraoperative	Glucose levels, Insulin requirement, wound infection and dehiscence	6
			C (*n* = 104)	No treatment		
Harding et al. (Harding et al. 2021)	2021	Cohort study	Dex (*n* = 173)	Dex 4–10 mg intraoperative	Hyperglycemia, glucose levels, wound infection	7
			C (*n* = 55)	No treatment		
O’Connell et al. (O'Connell et al. 2018)	2018	Cohort study	Dex (*n* = 77)	Dex intraoperative	Glucose levels	7
			C (*n* = 161)	No treatment		

*Dex* dexmethosone, *C* control, *RCT* randomized controlled trial

### Results of the meta-analysis

#### Effect of dexamethasone on intraoperative and/or postoperative glucose levels

Ten (Nazar et al. 2011; Tien et al. 2016; Nazar et al. 2009; Corcoran et al. 2021a; Purushothaman et al. 2018; Zhang et al. 2014; Shang et al. 2022; Wasfie et al. 2018; Egan et al. 2019; Corcoran et al. 2021b), six (Tien et al. 2016; Corcoran et al. 2021a; Purushothaman et al. 2018; Zhang et al. 2014; Shang et al. 2022; Wasfie et al. 2018), nine (Nazar et al. 2011; Tien et al. 2016; Corcoran et al. 2021a; Purushothaman et al. 2018; Zhang et al. 2014; Shang et al. 2022; Wasfie et al. 2018; Allen et al. 2020; O'Connell et al. 2018), six (Tien et al. 2016; Corcoran et al. 2021a; Shang et al. 2022; Egan et al. 2019; Harding et al. 2021; O'Connell et al. 2018) and two (Egan et al. 2019; O'Connell et al. 2018) studies reported glucose levels at baseline (preoperatively), intraoperatively (in the midterm of surgery), at the end of surgery, on postoperative day 1 (POD 1), and POD 2. The results in the trial by Corcoran TB 2020 (Corcoran et al. 2021a) were split in the forest plot, with Corcoran TB 2020(1) representing the comparison between dexamethasone 4 mg with control, and Corcoran TB 2020(2) representing the comparison between dexamethasone 8 mg with control. The results in the trial by Purushothaman AM 2018 (Purushothaman et al. 2018) were also split in the forest plot, with Purushothaman AM 2018(1) representing the comparison between dexamethasone 4 mg with control, and Purushothaman AM 2018(2) representing the comparison between dexamethasone 8 mg with control.

**Fig. 2:**
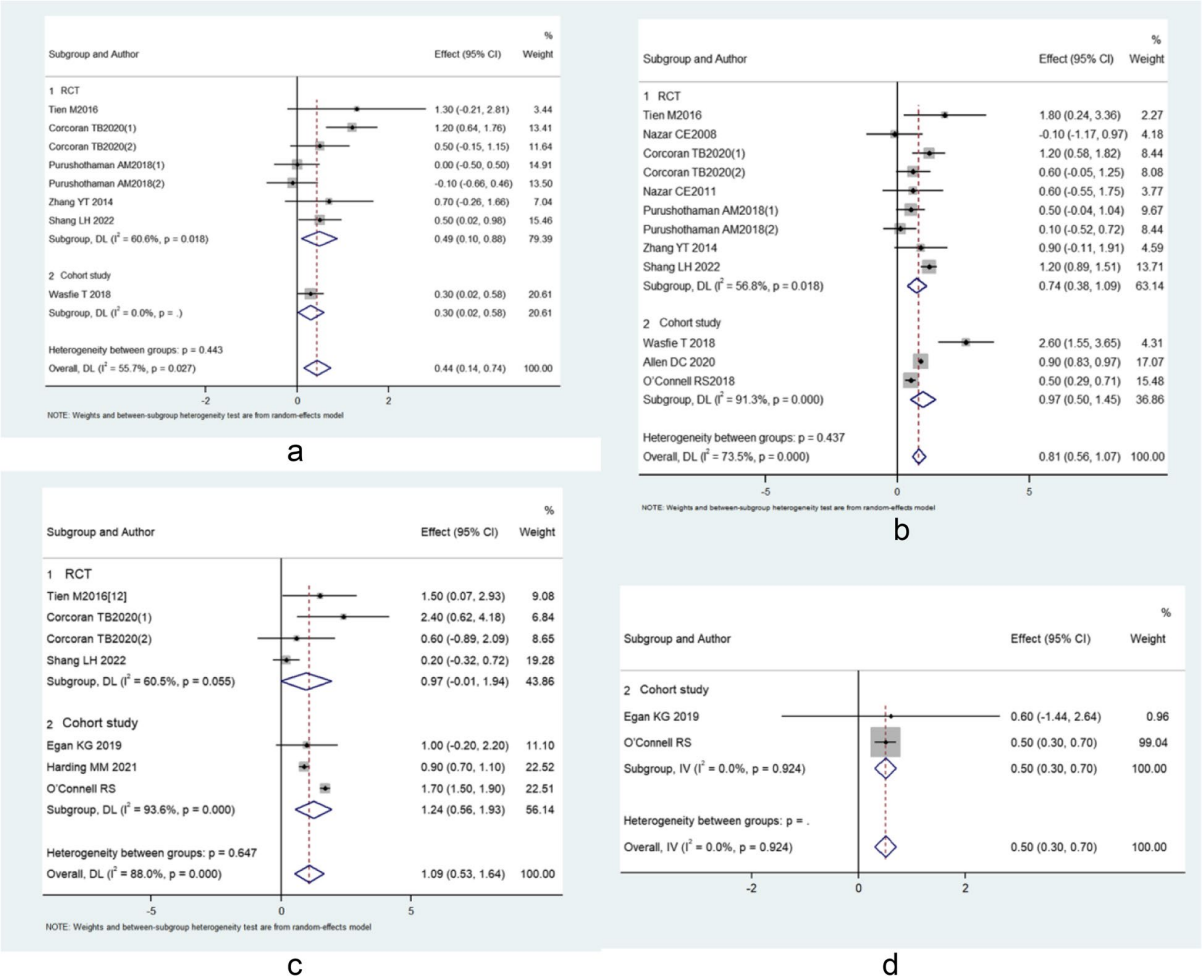
Forest plots of glucose response (dexamethasone versus control) **a** intraoperatively, **b** at the end of surgery, **c** on POD 1, **d** on POD 2

The results showed that there was no between-group difference in baseline glucose level (MD: -0.132, 95% CI: − 0.511-0.247, *I*^2^ = 87.3%, *P* = 0.496). Compared with control, dexamethasone could increase glucose level intraoperatively (MD: 0.439, 95% CI: 0.137–0.581, *I*^2^ = 55.7%, *P* = 0.004), at the end of surgery (MD: 0.815, 95% CI: 0.563–1.067, *I*^2^ = 73.5%, *P* = 0.000), on POD 1 (MD: 1.087, 95% CI: 0.534–1.640, *I*^2^ = 88%, *P* = 0.000), and on POD 2 (MD: 0.501, 95% CI: 0.301–0.701, *I*^2^ = 0%, *P* = 0.000) (Fig. 2). These results indicated that the increase of glucose level by dexamethasone was 0.439 mmol/L (7.90 mg/dL) intraoperatively, 0.815 mmol/L (14.67 mg/dL) at the end of surgery, 1.087 mmol/L (19.57 mg/dL) on POD 1, and 0.501 mmol/L (9.02 mg/dL) on POD 2.

**Fig. 3:**
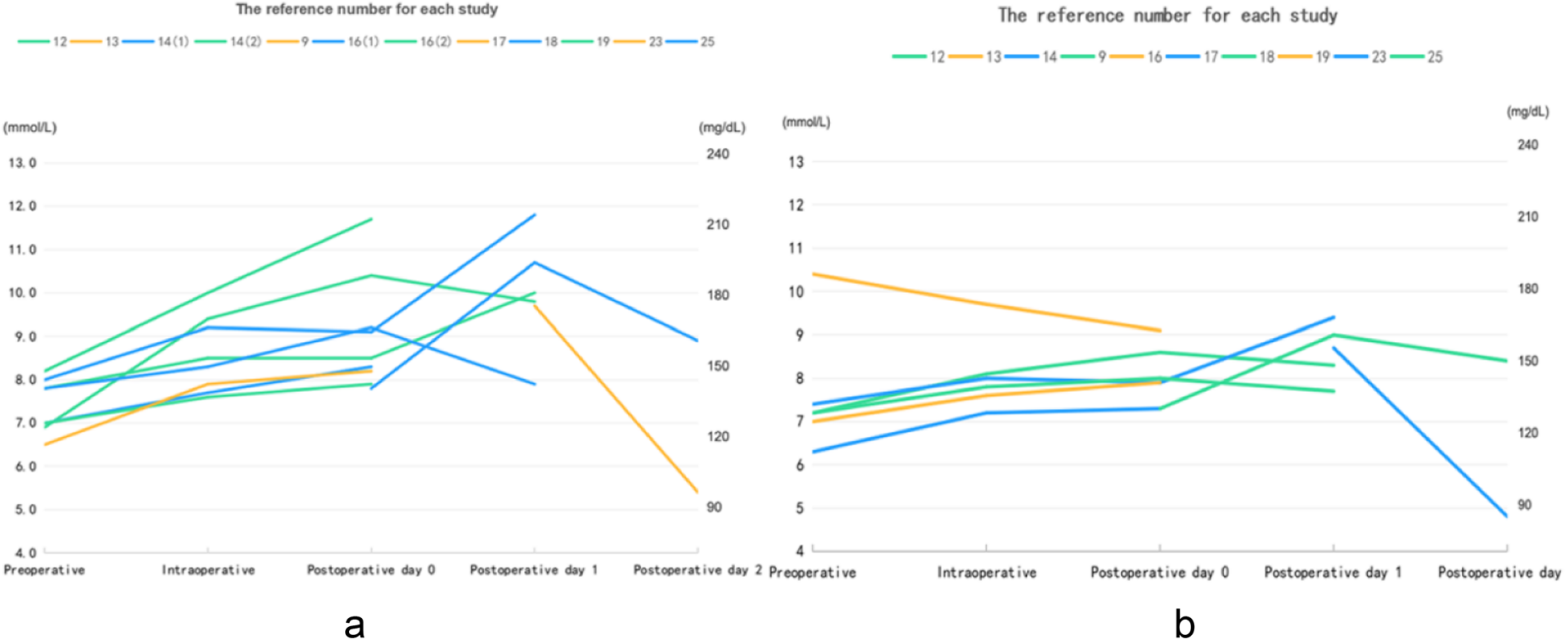
The trend of blood glucose changes. **a** dexamethasone group; **b** control group. 14(1): dexamethasone 4 mg; 14(2): dexamethasone 8 mg; 16(1): dexamethasone 4 mg; 16(2): dexamethasone 8 mg

The trend of blood glucose changes was presented in Fig. 3 and Suppl Table 1. It showed that the blood glucose reached its peak level within 24 hours of surgery in both dexamethasone and control groups, and then decreased afterwards.

Subgroup analysis for RCTs showed that dexamethasone could increase glucose level intraoperatively (MD: 0.489, 95% CI: 0.098–0.879, *I*^2^ = 60.6%, *P* = 0.014), and at the end of surgery (MD: 0.737, 95% CI: 0.381–1.093, *I*^2^ = 56.8%, *P* = 0.000), but there was no significantly differences between groups on POD 1 (MD: 0.966, 95% CI: − 0.007-1.939, *I*^2^ = 60.5%, *P* = 0.052).

Subgroup analysis for cohort studies showed that dexamethasone could increase glucose level at the end of surgery (MD: 0.972, 95% CI: 0.498–1.445, *I*^2^ = 91.3%, *P* = 0.000), on POD 1 (MD: 1.245, 95% CI: 0.559–1.930, *I*^2^ = 93.6%, *P* = 0.000), and on POD 2 (MD: 0.501, 95% CI: 0.301–0.701, *I*^2^ = 0%, *P* = 0.000).

#### Effect of dexamethasone on peak glucose level

**Fig. 4:**
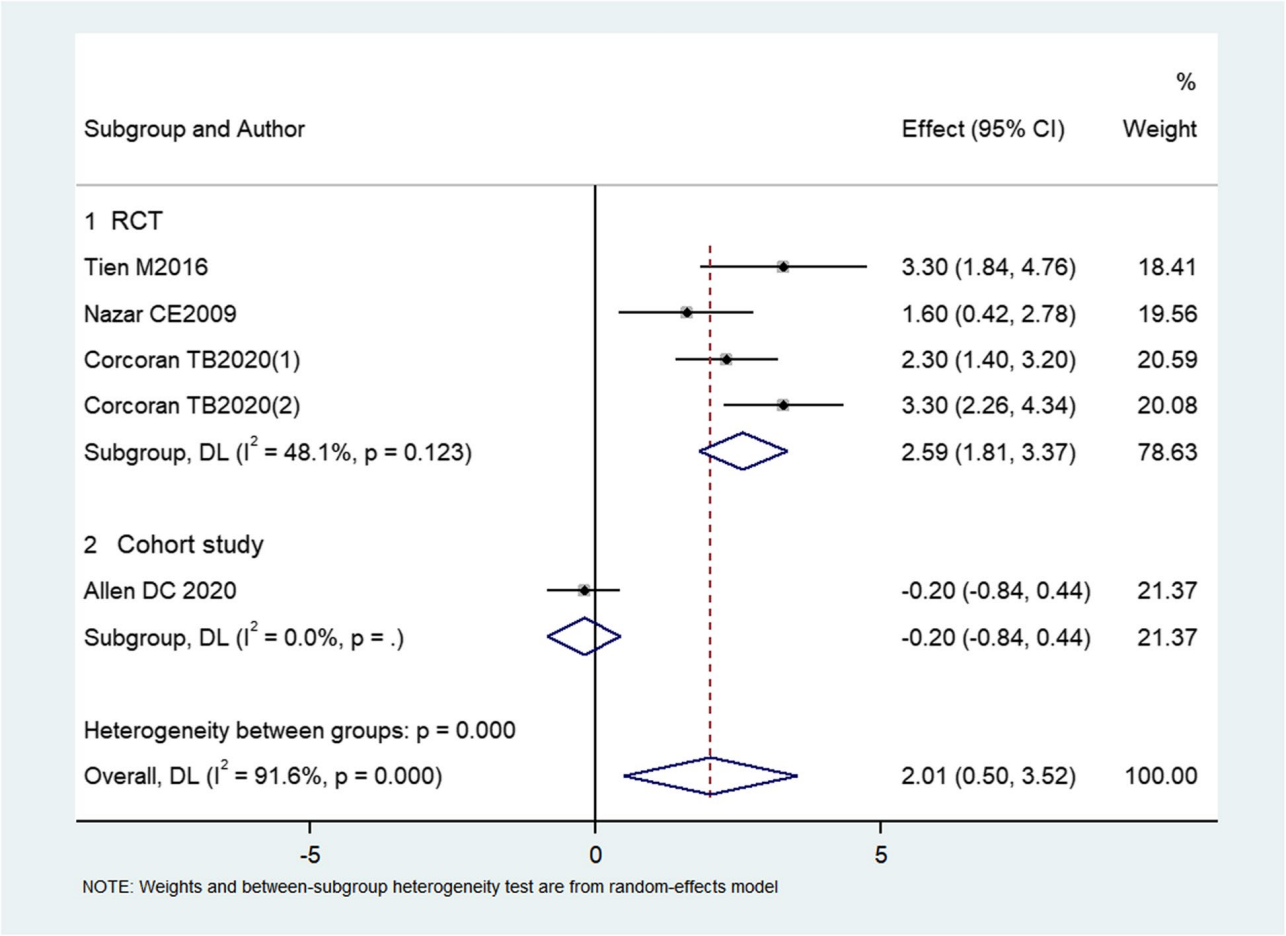
Forest plot of peak glucose level (dexamethasone versus control)

Three RCTs (Tien et al. 2016; Nazar et al. 2009; Corcoran et al. 2021a) and one cohort study (Allen et al. 2020) reported peak glucose level, which reached its peak level within 24 hours after surgery. The results in the trial by Corcoran TB2020 (Corcoran et al. 2021a) were split in the forest plot, with Corcoran TB2020(1) representing the comparison between dexamethasone 4 mg with control, and Corcoran TB2020(2) representing the comparison between dexamethasone 8 mg with control. The meta-analysis from all studies showed that dexamethasone increased peak glucose level compared with control (MD: 2.014, 95% CI: 0.503–3.525, *I*^*2*^ = 91.6%, *P* = 0.009) (Fig. 4), which indicated that the increase of peak blood glucose within 24 hours of surgery caused by dexamethasone was 2.014 mmol/L (36.252 mg/dL).

Subgroup analysis for RCTs showed that dexamethasone increased peak glucose level compared with control (MD: 2.566, 95% CI: 2.018–3.113, *I*^2^ = 48.1%, *P* = 0.000). The cohort study revealed no significant difference between groups (*P* = 0.539).

#### Effect of dexamethasone on insulin requirement

One RCT (Tien et al. 2016) investigated insulin requirement, there was no difference in the percentage of patients requiring insulin within 24 hours of surgery, but insulin dosage over 24 hours of surgery was higher in dexamethasone group than in control (*P* = 0.03). Three cohort studies (Wasfie et al. 2018; Allen et al. 2020; Harding et al. 2021) investigated insulin requirement, of which, 2 studies (Allen et al. 2020; Harding et al. 2021) reported postoperative insulin requirement, the other one (Wasfie et al. 2018) reported intraoperative requirement, and showed no difference between groups. The results of insulin requirement on the day of surgery (POD 0) were from 2 cohort studies (Allen et al. 2020; Harding et al. 2021), the result showed that there were no significant between-group differences on POD 0 (SMD: 1.735, 95%CI: − 0.556-4.027, *I*^2^ = 97.8%, *P* = 0.138); and those on POD1 were from only 1 study (Allen et al. 2020), which showed no between-group differences (*p* < 0.05).

#### Effect of dexamethasone on wound infection and dehiscence

**Fig. 5:**
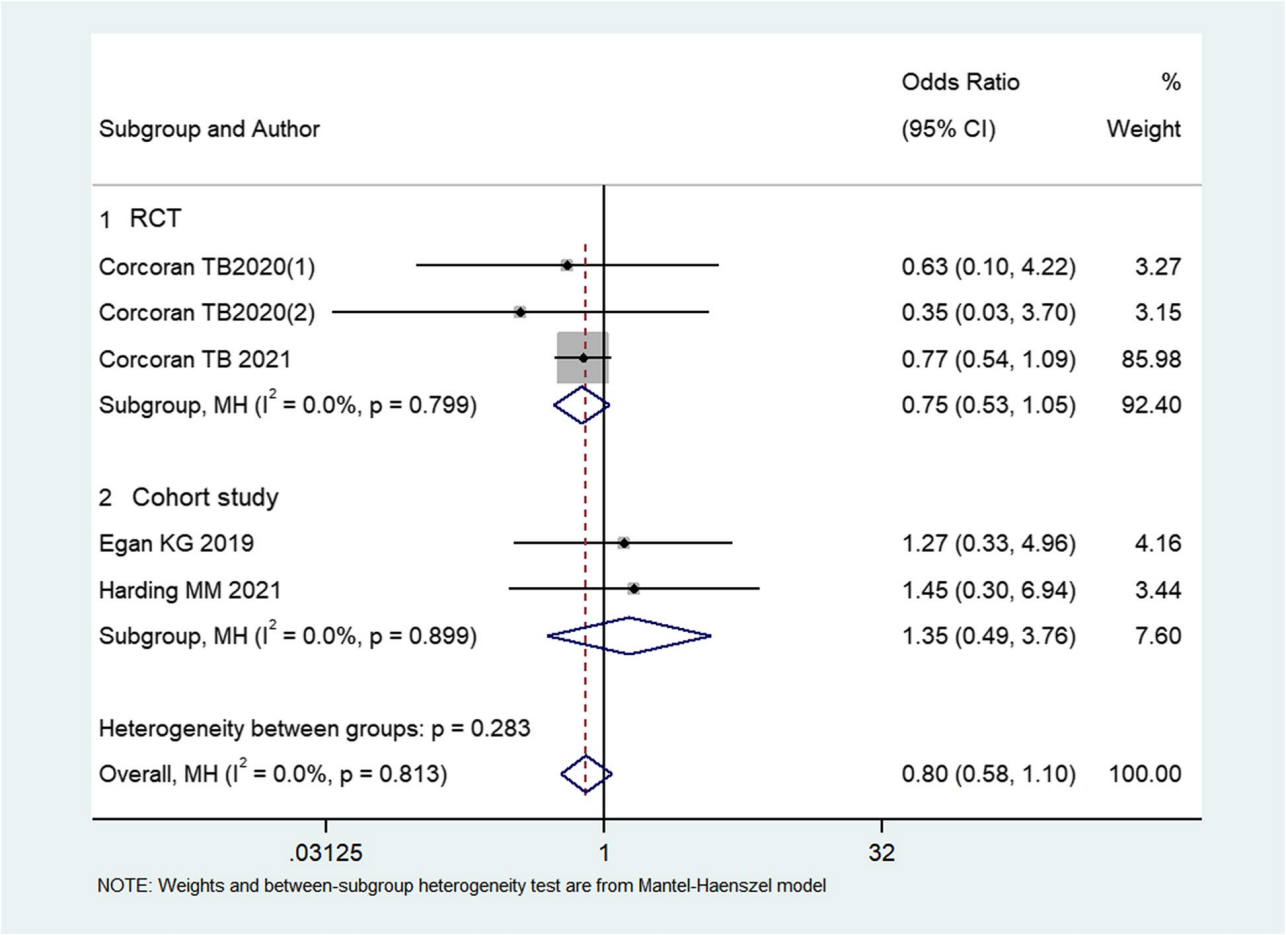
Forest plot of wound infection for dexamethasone versus control group

Two RCTs (Corcoran et al. 2021a; Corcoran et al. 2021b) and 2 cohort studies (Egan et al. 2019; Harding et al. 2021) compared wound infection, and the follow-up time ranged from 30 days to 90 days. The results in the trial by Corcoran TB2020 (Corcoran et al. 2021a) were split in the forest plot, with Corcoran TB2020(1) representing the comparison between dexamethasone 4 mg with control, and Corcoran TB2020(2) representing the comparison between dexamethasone 8 mg with control. The result from meta-analysis with fixed model method revealed that there was no between-group difference in wound infection (OR: 0.797, 95%CI: 0.578–1.099, *I*^2^ = 0%, *P* = 0.166) (Fig. 5).

Two RCTs (Corcoran et al. 2021a; Shang et al. 2022) compared wound dehiscence, no patient developed wound dehiscence in the two RCTs, so there were no statistical differences between groups. Only one cohort study (Egan et al. 2019) investigated wound dehiscence, the follow-up time was 90 days, and showed no between-group differences (*P* < 0.05).

#### Publication bias

Egger test showed that there was no significant publication bias between articles for all outcomes (*P* > 0.05), except the result of peak glucose level (*P* < 0.05), see Table 2.

**Table 2:**
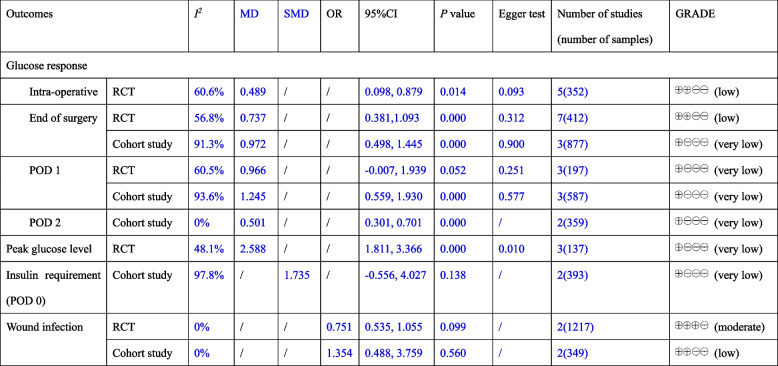
Summary of outcomes with evidence-based quality assessment

*MD* mean differences, *SMD* standardized mean differences, *OR* odds ratio, *CI* confidential index, *GRADE* Grading of Recommendations, Assessment, Development and Evaluation, *RCT* randomized controlled trial, *POD* postoperative day

#### GRADE for outcomes

The evidence-based quality from GRADE showed that the outcomes were in very low to moderate quality. The details were provided in Table 2.

## Discussion

This meta-analysis compares the effect of dexamethasone on blood glucose and wound healing in surgical patients with DM, and the results indicated that the blood glucose could be increased by dexamethasone, and dexamethasone had no effect on wound healing.

After intravenous administration, dexamethasone reaches its peak effect at 2–12 hours and lasted for 72 hours, but its effect on blood glucose persists less than 24 hours (Corcoran et al. 2021b). Some studies showed that dexamethasone increased blood glucose to a similar extent in diabetic patients to that in non-diabetic patients (Polderman et al. 2018; Purushothaman et al. 2018). One recent meta-analysis revealed that dexamethasone increased blood glucose within 12 hours after surgery in non-diabetic patients with a mean increase of 0.7 mmol/L(12.6 mg/dL), which seems not problem for non-diabetic patients in the clinic, and dexamethasone did not affect postoperative wound infection (Yue et al. 2017). In our meta-analysis, the blood glucose was increased by dexamethasone, it reached its peak level within 24 hours of surgery, and the increase of peak level was only 2.014 mmol/L (36.252 mg/dL) compared with control, the magnitude of glucose increase was even lower at each time point perioperatively [from 0.438 to 1.087 mmol/L (7.902 to 19.566 mg/dL)]. Furthermore, dexamethasone did not increase wound infection and influence wound healing. So, a single dose of dexamethasone can be safely used for PONV prophylaxis in diabetic patients.

In our meta-analysis, the dosage of dexamethasone in the included studies ranges from 4 to 12 mg. Dexamethasone with 4–10 mg is commonly used to prevent postoperative nausea and vomiting, and the effect of 8 mg or higher dose may be better (Gan et al. 2020; Corcoran et al. 2021b; Yue et al. 2017). The effect of increasing blood glucose is greater by larger dosage of dexamethasone (Corcoran and Edwards 2015; Low et al. 2015), but only few studies compared the differences between different dosages of dexamethasone, so we did not meta-analyze the related results.

This meta-analysis has some limitations. First, the dosages of dexamethasone and types of surgeries are different between the included RCTs or between cohort studies, which may have a potential impact on the results. Second, there are few studies reporting wound infections and other complications, and large RCTs are needed in the near future.

## Conclusion

This meta-analysis identified 9 RCTs and 7 cohort studies, investigated the effect of dexamethasone with antiemetic dosage on blood glucose and wound healing in diabetic surgical patients. Low to moderate evidence from RCTs and very low to low evidence from cohort studies suggested that the blood glucose was increased by a single dose of dexamethasone, the rise of peak glucose level was only 2.014 mmol/L (36.252 mg/dL), the increase of glucose level at each time point perioperatively was even lower, and dexamethasone had no effect on wound healing. Thus, dexamethasone with a single dose could be safely used for PONV prophylaxis in diabetic patients.

## Supplementary Information

**Additional file 1: Supplemental file 1.** Detailed search strategy in databases.

**Additional file 2: Suppl table 1.** The trend of mean glucose level [mg/dL(mmol/L)].

## Data Availability

All data generated or analyzed during this study are included in this published article and its supplemental information files.

## Abbreviations

DM: diabetes mellitus
PONV: postoperative nausea and vomiting
RCTs: randomized controlled trials
POD: postoperative day
MD: mean differences
SMD: standardized mean differences
CI: confidential index
OR: odds ratio
GRADE: Grading of Recommendations, Assessment, Development and Evaluation
