# Supplementary material for: The safety of perioperative dexamethasone with antiemetic dosage in surgical patients with diabetes mellitus: a systematic review and meta-analysis
Source: Perioper Med (Lond). 2023 Mar 8;12:4. doi: 10.1186/s13741-023-00293-4 (PMC9993727; doi:10.1186/s13741-023-00293-4)
Supplement: Supplementary file 1 — Additional file 1: Supplemental file 1. Detailed search strategy in databases. [file 13741_2023_293_MOESM1_ESM.docx]

Suppl table 1 The trend of mean glucose level [mg/dL(mmol/L)]

| Author | Groups | Preoperative | Intraoperative | Day 0 | Day 1 | Day 2 |
| --- | --- | --- | --- | --- | --- | --- |
| Tien etal [12] | Dex (8 mg) | 124.2 (6.9) | 169.2(9.4) | 187.2 (10.4) | 176.4 (9.8) | / |
|  | C | 129.6 (7.2) | 145.8(8.1) | 154.8 (8.6) | 149.4 (8.3) | / |
| Nazar etal [13] | Dex (8 mg) | 90 (5.0) | / | 126 (7.0) | / | / |
|  | C | 90 (5.0) | / | 127.8 (7.1) | / | / |
| Corcoran etal [14] | Dex (4mg) | 144 (8.0) | 165.6(9.2) | 163.8 (9.1) | 212.4 (11.8) | / |
|  | Dex (8mg) | 140.4 (7.8) | 153(8.5) | 153 (8.5) | 180 (10) | / |
|  | C | 133.2 (7.4) | 144(8.0) | 142.2 (7.9) | 169.2 (9.4) | / |
| Backes etal [15] | Dex (10mg) | / | / | / | / | / |
|  | C | / | / | / | / | / |
| Nazar et al [9] | Dex (8mg) | 100.8 (5.6) | / | 135 (7.5) | / | / |
|  | C | 109.8 (6.1) | / | 124.2 (6.9) | / | / |
| Purushothaman etal [16] | Dex (4mg) | 126 (7.0) | 138.6(7.7) | 149.4 (8.3) | / | / |
|  | Dex (8mg) | 12 6(7.0) | 136.8(7.6) | 142.2 (7.9) | / | / |
|  | C | 133.2 (7.4) | 138.6(7.7) | 140.4 (7.8) | / | / |
| Zhang et al [17] | Dex (10mg) | 117 (6.5) | 142.2(7.9) | 147.6 (8.2) | / | / |
|  | C | 113.4 (6.3) | 129.6(7.2) | 131.4 (7.3) | / | / |
| Shang et al [18] | Dex (0.11mg/kg) | 140.4 (7.8) | 149.4(8.3) | 165.6 (9.2) | 142.2 (7.9) | / |
|  | C | 129.6 (7.2) | 140.4(7.8) | 144 (8.0) | 138.6 (7.7) | / |
| Corcoran et al [26] | Dex (8mg) | 122.4 (6.8) | / | / | / | / |
|  | C | 118.8 (6.6) | / | / | / | / |
| Wasfie et al [19] | Dex (no detail) | 147.6 (8.2) | 180(10.0) | 210.6 (11.7) | / | / |
|  | C | 187.2 (10.4) | 174.6(9.7) | 163.8 (9.1) | / | / |
| Herbst etal [20] | Dex (no detail) | / | / | / | / | / |
|  | C | / | / | / | / | / |
| Godshaw et al [21] | Dex (6 or 12mg) | / | / | / | / | / |
|  | C | / | / | / | / | / |
| Allen et al [22] | Dex (8mg) | / | / | 140.4 (7.8) | / | / |
|  | C | / | / | 124.2 (6.9) | / | / |
| Egan et al [23] | Dex (4 or 10mg) | 154.8 (8.6) | / | / | 174.6 (9.7) | 97.2 (5.4) |
|  | C | 145.8 (8.1) | / | / | 156.6 (8.7) | 86.4 (4.8) |
| Harding et al [24] | Dex (4-10mg) | / | / | / | 163.8 (9.1) | / |
|  | C | / | / | / | 147.6 (8.2) | / |
| O'Connell et al [25] | Dex (no detail) | / | / | 140.4 (7.8) | 192.6 (10.7) | 160.2 (8.9) |
|  | C | / | / | 131.4 (7.3) | 162 (9.0) | 151.2 (8.4) |

Dex: dexmethosone; C: control
