# Supplementary material for: The safety of perioperative dexamethasone with antiemetic dosage in surgical patients with diabetes mellitus: a systematic review and meta-analysis
Source: Perioper Med (Lond). 2023 Mar 8;12:4. doi: 10.1186/s13741-023-00293-4 (PMC9993727; doi:10.1186/s13741-023-00293-4)
Supplement: Supplementary file 2 — Additional file 2: Suppl table 1. The trend of mean glucose level [mg/dL(mmol/L)]. [file 13741_2023_293_MOESM2_ESM.docx]

Supplemental file 1 Detailed **search strategy** in databases

| **Database** | **Search strategy** | **Number of studies** |
| --- | --- | --- |
| **Pubmed** | #1 diabetes mellitus[MeSH terms]  #2 (diabetes mellitus) OR (diabetic) OR (diabetes)  #3 (surgery) OR (surgical) OR (operation) OR (intraoperative) OR (perioperative)  #4 dexamethasone [MeSH terms]  #5 (dexamethasone) OR (steroids) OR (antiemesis) OR (antiemetic) OR (emesis) OR (emetic)  #6 (glucose) OR (glucose response) OR (hyperglycemia) OR (adverse event) OR (wound infection) OR (wound healing) OR (PONV) OR (postoperative nausea and vomiting)  #7 (#1 OR #2) AND #3 AND (#4 OR #5) AND #6 | **1460** |
| ****Web of Science**** | (AB=(diabetes mellitus) OR (diabetic) OR (diabetes)) AND (AB=(surgery) OR (surgical) OR (operation) OR (intraoperative) OR (perioperative) ) AND (AB=(dexamethasone) OR (steroids) OR (antiemesis) OR (antiemetic) OR (emesis) OR (emetic)) AND (AB=(glucose) OR (glucose response) OR (hyperglycemia) OR (adverse event) OR (wound infection) OR (wound healing) OR (PONV) OR (postoperative nausea and vomiting)) | **811** |
| ****Embase**** | ('diabetes mellitus'/exp OR diabetic:ti,ab OR diabetes:ti,ab) AND ('dexamethasone'/exp OR steroid:ti,ab OR antiemesis:ti,ab OR antiemetic:ti,ab OR emesis:ti,ab OR emetic:ti,ab) AND ('surgery'/exp OR surgical:ti,ab OR operation:ti,ab OR intraoperative:ti,ab OR perioperative:ti,ab) AND ('glucose'/exp OR 'glucose response':ti,ab OR hyperglycemia:ti,ab OR 'wound healing':ti,ab OR 'wound infection':ti,ab OR 'adverse event'/exp) | **1597** |
| ****Cochrane Library**** | #1 MeSH descriptor:[diabetes mellitus] explore all trees  #2 (diabetes mellitus )OR (diabetic) OR (diabetes)  #3 (surgery) OR (surgical) OR (operation) OR (intraoperative) OR (perioperative)  #4 MeSH descriptor:[dexamethasone] explore all trees  #5 (dexamethasone) OR (steroids) OR (antiemesis) OR (antiemetic) OR (emesis) OR (emetic)  #6 (glucose) OR (glucose response) OR (hyperglycemia) OR (adverse event) OR (wound infection) OR (wound healing) OR (PONV) OR (postoperative nausea and vomiting)  #7 (#1 OR #2) AND #3 AND (#4 OR #5) AND #6 | **397** |
| ****CNKI**** | （篇关摘：糖尿病（模糊））AND（篇关摘：地塞米松（模糊））AND（篇关摘：手术（模糊）） | **176** |
